# Fibrin Scaffolds Perfused with Transforming Growth Factor-β1 as an In Vitro Model to Study Healthy and Tendinopathic Human Tendon Stem/Progenitor Cells

**DOI:** 10.3390/ijms25179563

**Published:** 2024-09-03

**Authors:** Maria Camilla Ciardulli, Joseph Lovecchio, Ornella Parolini, Emanuele Giordano, Nicola Maffulli, Giovanna Della Porta

**Affiliations:** 1 Translational Nanomedicine Laboratory, Department of Medicine, Surgery and Dentistry, University of Salerno, Via S. Allende 43, 84081 Baronissi, Italy; mciardulli@unisa.it; 2 School of Science and Engineering, Reykjavík University, 102 Reykjavík, Iceland; joseph.lovecchio@unibo.it; 3 Institute of Biomedical and Neural Engineering, Reykjavik University, 102 Reykjavík, Iceland; 4 Department of Life Science and Public Health, Università Cattolica del Sacro Cuore, 00168 Rome, Italy; ornella.parolini@unicatt.it; 5 Fondazione Policlinico Universitario “Agostino Gemelli” IRCCS, Università Cattolica del Sacro Cuore, 00136 Rome, Italy; 6 Laboratory of Cellular and Molecular Engineering “Silvio Cavalcanti”, Department of Electrical, Electronic and Information Engineering “Guglielmo Marconi” (DEI), University of Bologna, 47522 Cesena, Italy; emanuele.giordano@unibo.it; 7 Advanced Research Center on Electronic Systems (ARCES), University of Bologna, 40126 Bologna, Italy; 8 Department of Trauma and Orthopaedics, Faculty of Medicine and Psychology, Sant’ Andrea Hospital, Sapienza University, 00189 Rome, Italy; nicola.maffulli@uniroma1.it; 9 Interdepartment Centre BIONAM, University of Salerno, Via Giovanni Paolo II 132, 84084 Fisciano, Italy

**Keywords:** tenogenesis, tissue engineering, human tendon/stem progenitor cells, 3D advanced in vitro model, cytokines, dynamic culture, perfusion, human transforming growth factor-β1

## Abstract

A limited understanding of tendon cell biology in healthy and pathological conditions has impeded the development of effective treatments, necessitating in vitro biomimetic models for studying tendon events. We established a dynamic culture using fibrin scaffolds, bioengineered with tendon stem/progenitor cells (*h*TSPCs) from healthy or diseased human biopsies and perfused with 20 ng/mL of human transforming growth factor-β1 for 21 days. Both cell types showed long-term viability and upregulated Scleraxis (SCX-A) and Tenomodulin (TNMD) gene expressions, indicating tenogenic activity. However, diseased *h*TSPCs underexpressed collagen type I and III (COL1A1 and COL3A1) genes and exhibited lower SCX-A and TNMD protein levels, but increased type I collagen production, with a type I/type III collagen ratio > 1.5 by day 14, matching healthy cells. Diseased *h*TSPCs also showed constant high levels of pro-inflammatory cytokines, such as IL-8 and IL-6. This biomimetic environment is a valuable tool for studying tenogenic and inflammatory events in healthy and diseased tendon cells and identifying new therapeutic targets.

## 1. Introduction

Tendons are well-organized, dense connective tissues that respond and adapt to the transmission of contraction forces by muscles to the skeleton, allowing motion and maintenance of posture [1]. Tendon injuries, also known as tendinopathies, are becoming more common as populations age and participation in sports/leisure activities increases. In tendinopathy, the pathological changes can be interpreted as a failure in the homeostatic response of the tendon to adapt to altered mechanical loading, resulting in permanent changes in the native tendon structures and mechanics [1,2]. The hypovascular and hypocellular nature of tendon tissues limits their healing capacity and contributes to the loss of functionality and propensity to reinjury [3]. Since tendon healing is a complex coordinated event governed by the active interactions between resident cells and chemical and mechanical external stimuli [4], it is important to identify key molecular and cellular processes involved in the progression of tendinopathies and the tendon’s response to them to develop effective therapeutic strategies and drive the tissue towards regeneration [5].

Despite the considerable progress made in identifying several important players in tendon biology, neither the ontogeny of the tenogenic lineage nor an accurate analysis of the heterogeneous cell population resident in tendon tissue has been provided [5]. For this reason, it becomes of primary importance to unequivocally identify the role of resident or stem cells in the establishment of tenogenic markers and to study their response to bioactive molecules, such as growth factors [6,7] and biomechanical stimuli [8]. Traditionally, tendons were believed to contain only tenocytes, which were responsible for the maintenance and repair of the tissue [9]. Resident cells, which possess multipotency, self-renewal and clonogenicity properties, were identified as stem cells in animals and humans [10,11]. Tendon stem cells, commonly referred to as tendon stem/progenitor cells (TSPCs) are situated in a distinct niche consisting of a unique extracellular matrix (ECM) [2]. Unlike other unknown stem cell niches, the TSPC niche is described, given the great abundance of tendons in ECM components and the lower number of cells compared with other tissues, as mostly made and regulated by ECM components, such as collagens, large proteoglycans, and small, leucine-rich proteoglycans, which function as lubricators and organizers for collagen fibril assembly [11]. Recent advances have proved that TSPCs facilitate tendon healing through three mechanisms: regulating the inflammatory response, enhancing tenocyte proliferation, and expediting collagen production, while regulating ECM remodeling [12]. Therefore, TSPCs are considered a suitable cell source for tendon repair, even if, to the best of our knowledge, little research has been conducted on human TSPCs in the last 10 years [10,13,14,15,16,17,18].

Given the key role of the ECM in governing cell functions in tendons, recent advances in the tissue engineering area have focused on the development of bioengineered scaffolds. Indeed, compared to conventional two-dimensional (2D) culture systems, the major strengths of three-dimensional (3D) cultures are enhanced intercellular communications and a similarity to in vivo physiological conditions [19]. Both natural (e.g., collagen, chitosan, fibrin) [20,21,22] and synthetic [e.g., poly (L-lactic-co-glycolic acid) (PLGA), polycaprolactone (PCL), polyethylene terephthalate (PET), polyethylene glycol (PEG)] [23,24,25] polymers have been used in tendon tissue engineering approaches to constitute aligned scaffolds or hydrogels. Even though hydrogels do not have structured morphologies and high biomechanical strength, they have been widely used because of their excellent plasticity and biocompatibility [26]. Among the different hydrogels, fibrin gels have been recently proposed for creating biomimetic tissue constructs in vitro, promoting greater collagen synthesis (than collagen-based hydrogels) [27]. Moreover, after cell culture, fibrin gel naturally shrinks and forms a fiber-like structure and it can be used for a 3D model of tenogenesis [19]. However, to date, an investigation of the behavior of human TSPCs when cultured in fibrin scaffolds has never been conducted.

To produce functionalized tissues in vitro and to overcome the limitations associated with static culture environments, tendon tissue engineering combines bioengineered scaffolds with proper biochemical and physical stimuli using bioreactors. Several custom-made bioreactors have been developed for tendon tissue engineering [28], essentially based on cyclic stretching [29] and/or perfusion [30]. They can strictly control the local microenvironment by providing nutrients for cell metabolism and turnover and delivering mechanical stimuli that regulate the construct of homeostasis in vitro [28]. Moreover, they better support cell alignment and differentiation and the ECM deposition [28]. Unfortunately, despite the acknowledged importance of mechanotransduction mechanisms for tendon function and repair, so far, only a few studies have reported on the effects of mechanical stimulation on TSPCs. Most of these studies have exposed cells to uniaxial or biaxial cyclic strain, enhancing tenogenic differentiation [17,31], but the tenogenic properties of TSPCs have yet to be investigated in regard to perfusion conditions.

Bioreactor systems can also provide an appropriate biochemical environment to stimulate tenogenic events and ECM synthesis [28]. Numerous bioactive molecules are involved in orchestrating the cellular response during tendon repair. A variety of growth factors are markedly upregulated following tendon injuries and are active at multiple stages of the healing process, including insulin-like growth factor-I (IGF-I) [32], transforming growth factor-β (TGF-β), the basic fibroblast growth factor (bFGF), the platelet-derived growth factor (PDGF) [32], the vascular endothelial growth factor (VEGF), the bone morphogenetic protein (BMP), and the connective tissue growth Factor (CTGF) [5]. In vivo, TGF-β signalling was found to be a pivotal pathway in the specification and differentiation of TSPCs during growth and development [33] and for their recruitment into degenerative tendons [34]. The TGF-β1/Smad2/3 pathway, in particular, is involved in tendon injury recovery by enhancing the synthesis of type I and III collagen [35,36]. Moreover, the stimulation of cells with TGF-β in bioreactors was shown to increase the expression of tenogenic markers, matrix production and organization, and the mechanical properties [37,38].

We have previously established and characterized two clinically relevant cell types, TSPCs isolated from tendinopathic human Achilles tendon biopsies (referred to as pathological *h*TSPCs) and TSPCs harvested from semitendinosus biopsies from healthy donors (healthy *h*TSPCs) [39]. Moreover, the *h*TSPCs displayed multipotency potential and a sustained proliferative rate [40], but with different morphology and gene expression modifications in terms of their dependence on the healthy or pathological conditions. Moreover, the characteristic overexpression of pro-inflammatory cytokines was detected in pathological cells [39]. In this study, we aimed to develop an in vitro model for the study of tenogenic events, adopting a dynamic culture involving a three-dimensional (3D) fibrin scaffold, bioengineered with healthy or pathological human TSPCs. The 3D culture was maintained for 21 days, under perfusion provided by a custom-made bioreactor, in a medium supplemented with human TGF-β1 at 20 ng/mL. In this setting, we analyzed and compared both the gene and protein expression of tenogenic markers by healthy and pathological *h*TSPCs, applying a range of techniques, including real-time quantitative polymerase chain reaction (RT-qPCR) tests, Immunofluorescence, and Sirius Red staining. We further investigated the gene expression profiles for pro-inflammatory and anti-inflammatory cytokines of both cell types and the secreted cytokines in the culture medium were quantified by an immunobead-based multiplex assay.

## 2. Results

### 2.1. Three-Dimensional Fibrin Scaffold Characterization

**Figure 1:**
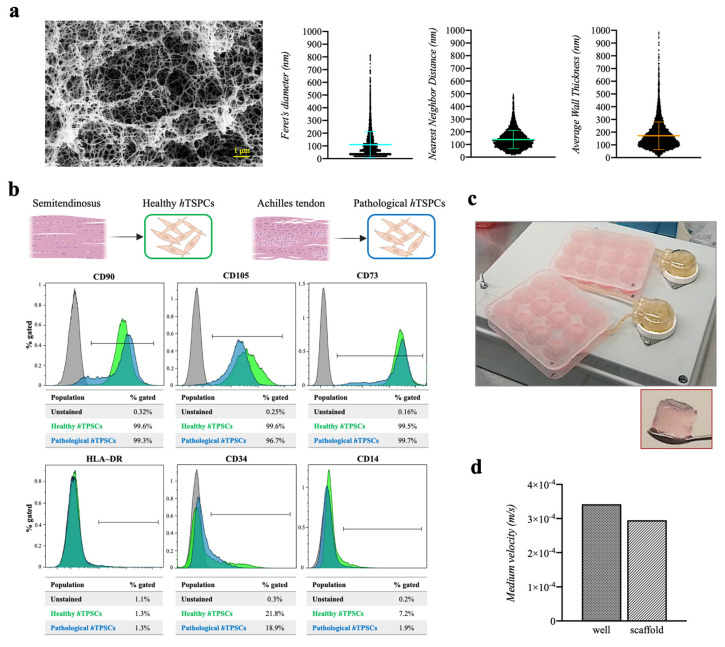
Fibrin scaffold characterization, flow cytometry analysis of healthy and pathological *h*TSPCs and dynamic culture setting. Field emission scanning electron microscopy (FE-SEM) images of aerogels, showing their internal structure and morphology; Feret’s diameter (nm), nearest neighbor distance (nm), and average wall thickness (nm) values, calculated using ImageJ software (rel.1.52p) (**a**). The *h*TSPCs were harvested from semitendinosus and Achilles tendon biopsies in humans. The normalized cell count histograms display the surface marker expression (CD45, CD90, CD73, CD105, HLA-DR, CD34, and CD14) for healthy and pathological *h*TSPCs; n = 4 (biological replicates) (**b**). Both cell types were used to bioengineer 3D fibrin scaffolds, cultured in a perfusion bioreactor that allows a constant *h*TGF-β1 supplemented medium flow rate of 1 mL/min (**c**). Culture medium velocity (m/s) values in the bioreactor wells and within the scaffold structure (**d**).

A fibrinogen concentration of 50 mg/mL was used to produce fibrin scaffolds as support for the cell culture, based on our previous optimization [41]. To observe the internal scaffold morphology using field emission scanning electron microscopy (FE-SEM), hydrogels were converted into aerogels using a dense gas drying process, to avoid the natural shrinkage and collapse of the 3D hydrated system (that normally occurs in lyophilization or drying), as previously described [42]. The scaffolds showed a porous structure, with a mean Feret’s diameter of 110 nm. The analysis also revealed a mean distance between the six nearest neighbors (pores) of 139 nm, indicating an interconnected porous structure. Moreover, thin fibrin meshes were observed, with a mean pore wall thickness of 172 nm (Figure 1a). These data described the fibrin scaffolds as a microenvironment with a high degree of void space available for cell distribution and viability.

### 2.2. The hTSPC Immunophenotyping and 3D Culture Establishment

Healthy and pathological *h*TSPCs were extracted from human biopsies, adopting a method we described elsewhere [39]. Immunophenotyping of both cell types was investigated by flow cytometry. The analysis indicated that both healthy and pathological tissue-derived *h*TSPCs expressed positive mesenchymal stem cell surface markers (CD90, CD73) and CD105 (a TGF-β receptor accessory molecule), but were negative for HLA-DR, CD34, and CD14 (Figure 1b). Then, both healthy and pathological *h*TSPCs were used to bioengineer fibrin scaffolds, and were cultured in dynamic conditions for up to 21 days using a custom-made perfusion bioreactor, with a constant flow rate of 1 mL/min [43] (Figure 1c). Medium velocity was calculated both in the wells and through the 3D scaffold, resulting in about 3.42 × 10^−4^ m/s in the culture chamber and 2.95 × 10^−4^ m/s in the 3D environment, due to the resistance offered by the scaffold mesh (Figure 1d). The calculated values suggested a good level of mass transport of nutrients through the 3D culture [44,45].

### 2.3. Perfusion-Based Dynamic Culture Assures Long-Term Viability

**Figure 2:**
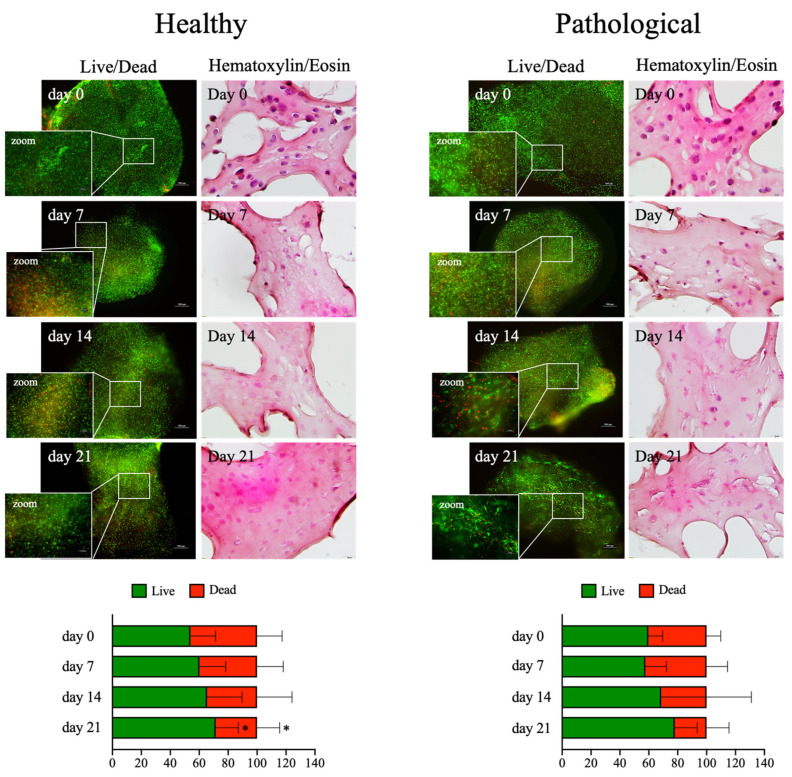
Live and dead assays and Hematoxylin and Eosin staining of healthy and pathological *h*TSPCs. Cells were cultured within the 3D fibrin scaffold in a medium supplemented with *h*TGF-β1 under perfusion for up to 21 days. Viable cells appear in green and non-viable cells in red. The fluorescence signal was quantified at different time points (0, 7, 14, 21 days) using ImageJ software (rel.1.52p). Scale bar: 500 μm, 200 μm for magnification (L&D); 50 μm (H&E). Data are shown as mean ± SD; * *p* ≤ 0.05 vs. day 14; n = 4 (biological replicates).

The cell viability within fibrin scaffolds was assessed by live and dead assays at different time points (0, 7, 14, and 21 days) over the culture period. Almost 80% of the loaded cells were represented by live cells throughout the culture for healthy and pathological groups (Figure 2). Furthermore, the fibrin scaffolds maintained their 3D shape, with living cells always homogenously distributed within the hydrogel matrix, as also documented by the histological evaluation of the 3D scaffold culture slices using Hematoxylin and Eosin staining (Figure 2). The shape of the pathological *h*TSPCs was unexpected, which showed an elongated shape on days 14 and 21; this shape was not observed for healthy cells, which maintained their typical spherical form within the 3D environment (see zoomed in fields in Figure 2).

### 2.4. Healthy and Pathological hTSPCs Display Different Collagen Gene Expression

**Figure 3:**
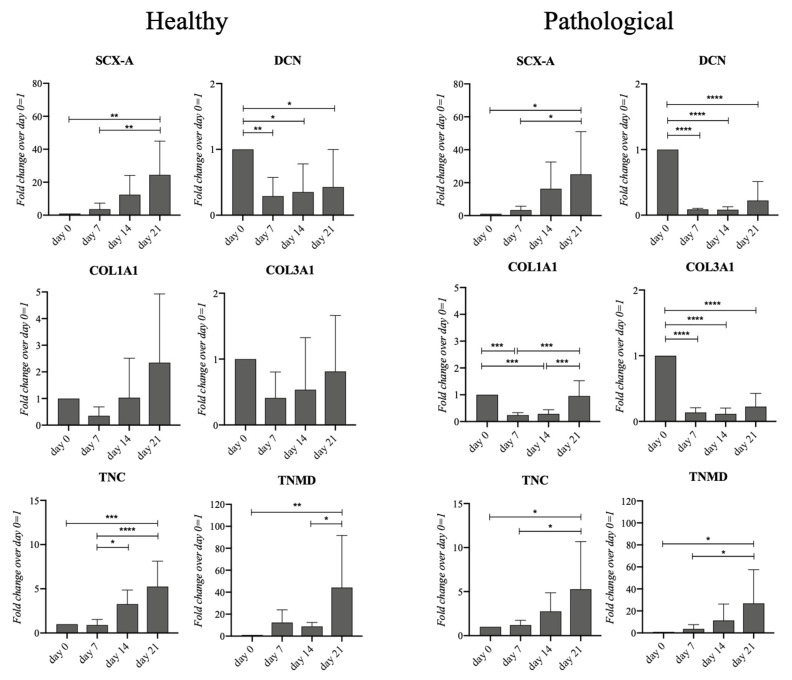
Gene expression profiles for tenogenic markers of healthy and pathological *h*TSPCs. Both cell populations were cultured within the 3D fibrin scaffold in a medium supplemented with *h*TGF-β1 under perfusion for up to 21 days. Different tenogenic markers (SCX-A, DCN, COL1A1, COL3A1, and TNC) were monitored by RT-qPCR at fixed time points (0, 7, 14, 21 days). Relative quantification for each mRNA gene expression normalized to endogenous GAPDH (internal control) was calculated using the 2^−ΔΔCt^ method and presented as fold change over day 0 (*h*TSPCs just after inclusion in the fibrin scaffold) = 1. * *p* < 0.05; ** *p* < 0.01; *** *p* < 0.005; **** *p* < 0.001 vs. day 0; n = 4 (biological replicates).

The tenogenic events of both healthy and pathological cells in our 3D perfused system were investigated using gene expression profiling of the tendon-related markers (SCX-A, DCN, COL1A1, COL3A1, TNC, and TNMD) (Figure 3). Healthy *h*TSPCs showed a significant upregulation of SCX-A (25-fold, *p* < 0.01), TNC (5-fold, *p* < 0.005), and TNMD (40-fold, *p* < 0.01) on day 21, while DCN appeared slightly, but significantly, downregulated (0.6-fold, *p* < 0.05). Similarly, for pathological *h*TSPCs, the SCX-A (25-fold, *p* < 0.05), TNC (5-fold, *p* < 0.05), and TNMD (25-fold, *p* < 0.05) expression significantly increased on day 21, while DCN decreased (0.8-fold, *p* < 0.001). What differed between the two cell types were the COL1A1 and COL3A1 expression levels. Indeed, healthy cells did not display significant variations in terms of both COL1A1 and COL3A1 expression throughout the culture, whereas, in pathological cells, COL1A1 expression was downregulated on days 7 and 14 (0.8-fold, *p* < 0.005) and returned to the basal level on day 21, while a constant and significant downregulation of COL3A1 (0.8-fold, *p* < 0.001) occurred throughout the culture.

### 2.5. Healthy and Pathological hTSPCs Show Differences in Tendon-Related Protein Expression

**Figure 4:**
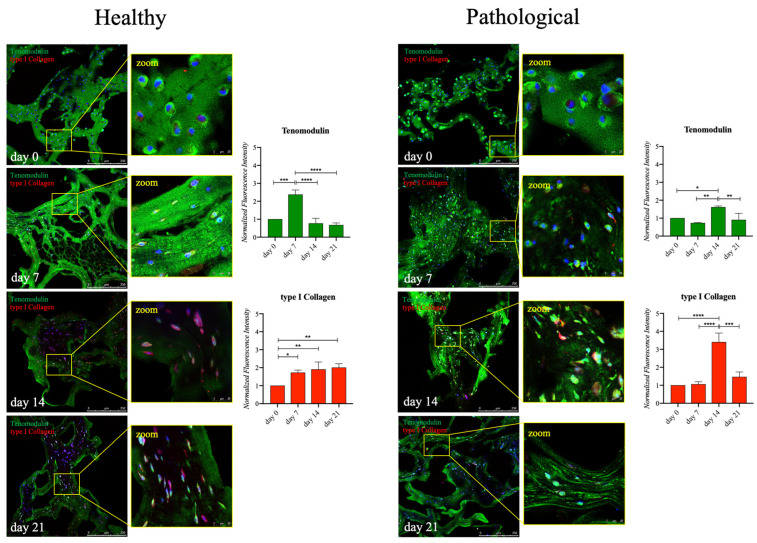
Immunofluorescence analysis of Tenomodulin and type I collagen proteins in healthy and pathological *h*TSPCs. Both cell populations were cultured within the 3D fibrin scaffold in a medium supplemented with *h*TGF-β1 under perfusion for up to 21 days. Signal intensity at each time point (0, 7, 14, 21 days) underwent semiquantitative analysis using ImageJ software (rel.1.52p) and normalized by the cell number (e.g., by the number of cell nuclei revealed by DAPI staining). Scale bar: 250 μm (magnification: 25 μm). Data are shown as mean ± SD. * *p* < 0.05; ** *p* < 0.01; *** *p* < 0.005; **** *p* < 0.001 vs. day 0; n = 4 (biological replicates).

To further investigate the behavior of both healthy and pathological *h*TSPCs concerning tenogenic events, tendon-related proteins, such as Scleraxis-A, Tenomodulin, and type I and type III collagen, were monitored by semiquantitative immunofluorescence. In the ECM of healthy cells, Tenomodulin was detectable from day 0, with a signal that became brighter on day 7 (2.3-fold increase, *p* < 0.005) and went below baseline levels on days 14 and 21. Whereas, pathological cells, whose matrix seemed highly disorganized, showed an increased deposition of Tenomodulin on day 14 (1.7-fold, *p* < 0.05). COL1A1 mRNA levels did not show significant variations for healthy *h*TSPCs when analysed by the RT-qPCR, but a constant increment in the type I collagen protein signal was observed throughout the culture, reaching a 2-fold increase (*p* < 0.01) on day 21. Conversely, for pathological *h*TSPCs, a very high and significant expression (3.5-fold, *p* < 0.001) was found in this regard only on day 14 (Figure 4).

**Figure 5:**
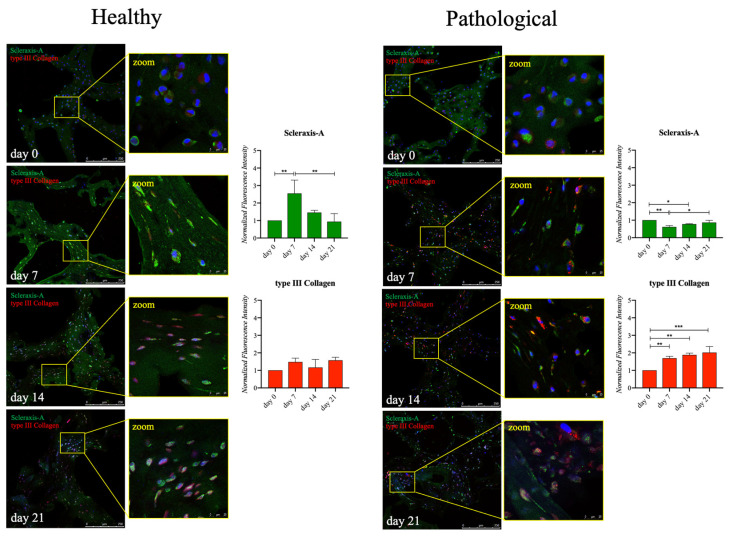
Immunofluorescence analysis of Scleraxis-A and type III collagen proteins in healthy and pathological *h*TSPCs. Both cell populations were cultured within the 3D fibrin scaffold in a medium supplemented with *h*TGF-β1 under perfusion for up to 21 days. Signal intensity at each time point (0, 7, 14, 21 days) underwent semiquantitative analysis using ImageJ software (rel.1.52p) and normalized by cell number (e.g., by the number of cell nuclei revealed by DAPI staining). Scale bar: 250 μm (magnification: 25 μm). Data are shown as mean ± SD. * *p* < 0.05; ** *p* < 0.01; *** *p* < 0.005 vs. day 0; n = 4 (biological replicates).

Regardless of the similar increasing trend of SCX-A observed by the RT-qPCR analysis, healthy and pathological *h*TSPCs showed different profiles of protein expression. Healthy *h*TSPCs had a 2.5-fold increase (*p* < 0.01) in Scleraxis-A expression on day 7, followed by a constant decrease over days 14 and 21. In contrast, for pathological *h*TSPCs, a slight but significant reduction (0.5-fold, *p* < 0.01) was measured between day 0 and day 7, then a constant increase was evidenced until day 21, but without exceeding the baseline values. Consistently with the RT-qPCR data, no significant differences were measured for type III collagen expression throughout the culture period in healthy cells. Instead, in pathological cells, opposite to the gene expression profile, type III collagen expression significantly rose over time, with a 2-fold increase (*p* < 0.005) on day 21 (Figure 5).

### 2.6. Pathological hTSPCs Show a Distinct Expression of Collagen Subtypes

**Figure 6:**
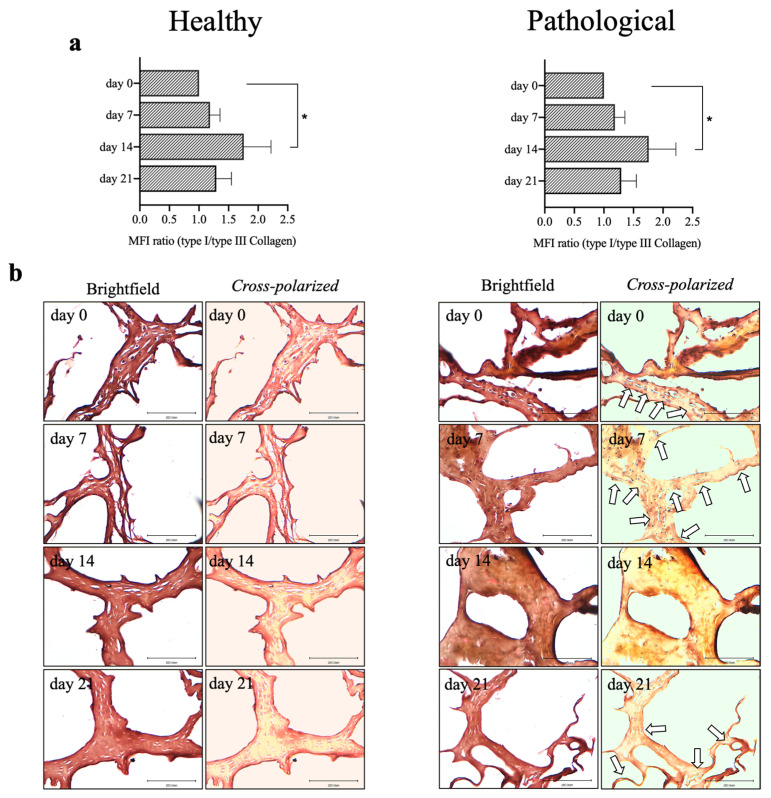
Analysis of type I and type III collagen protein expression in healthy and pathological *h*TSPCs. Type I/type III collagen protein ratio was calculated using mean fluorescence intensity (MFI) using ImageJ software (rel.1.52p) based on type I and type III collagen immunofluorescence data. The average intensities of three images taken from different sections of each patient (at each time point) were used for the calculation of the MFI (**a**). Sirius Red staining and polarized light images at different time points for healthy and pathological *h*TSPCs cultured within the 3D fibrin scaffold in a medium supplemented with *h*TGF-β1 under perfusion. Type I collagen fibers appear bright yellow and a small portion of type III collagen fibers are fine green (white arrows) (**b**). Scale bar: 200 μm. The ratio of type I to type III collagen is displayed as mean ± SD. * *p* < 0.05; n = 4 (biological replicates).

To better investigate the differential expression of collagen subtypes by healthy and pathological *h*TSPCs in our culture conditions, fluorescence intensities were used to estimate the ratio of type I to type III collagen. On day 14, the pathological cells exhibited the same type I/type III collagen ratio (>1.5) as healthy cells (*p* < 0.005). Whereas, on days 7 and 21, the ratio was higher in the healthy *h*TSPCs (>1), compared to the pathological *h*TSPCs (<1) (Figure 6a). The different expression patterns of the collagen subtypes were also documented through histological evaluation of the 3D scaffold culture slices using Sirius Red staining. Indeed, the healthy *h*TSPCs showed a prevalent deposition of type I collagen in the ECM, while type III collagen was more abundant in the ECM of pathological *h*TSPCs, except for day 14 (Figure 6b).

### 2.7. Pathological hTSPCs Exhibit Reduced Overall Cytokine Expression

**Figure 7:**
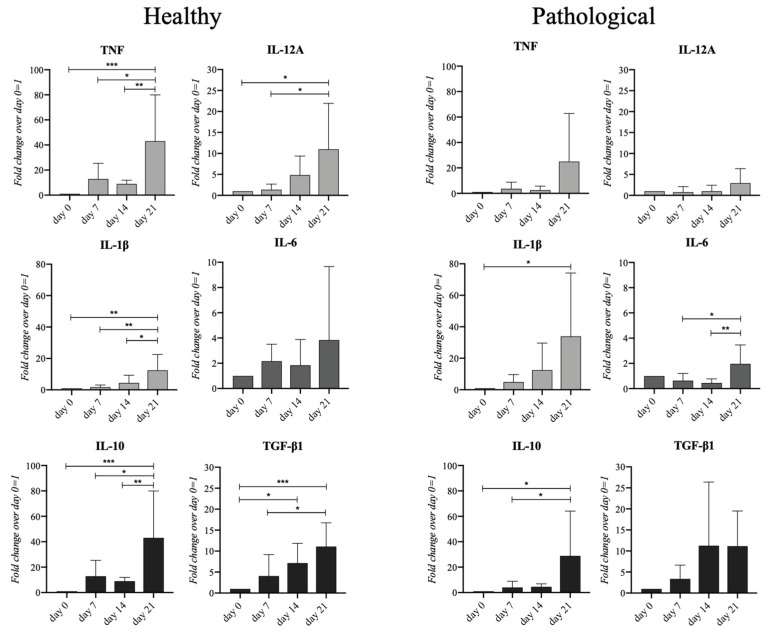
Gene expression profiles for pro-inflammatory and anti-inflammatory cytokines in healthy and pathological *h*TSPCs. Both cell populations were cultured within the 3D fibrin scaffold in a medium supplemented with *h*TGF-β1 under perfusion for up to 21 days. Pro-inflammatory (TNF, IL-12A, IL-1β, IL-6) and anti-inflammatory (IL-6, IL-10, TGF-β1) cytokines were monitored by RT-qPCR. Relative quantification of each mRNA gene expression normalized to endogenous GAPDH (internal control) was calculated using the 2^−ΔΔCt^ method and presented as the fold change during day 0 (hTSPCs just after inclusion in the fibrin scaffold) = 1. * *p* < 0.05; ** *p* < 0.01; *** *p* < 0.005 vs. day 0; n = 4 (biological replicates).

Cytokine expression throughout tenogenic events was monitored. Healthy *h*TSPCs showed a significant upregulation of pro-inflammatory cytokines, namely TNF (40-fold, *p* < 0.005), IL-12A (10-fold, *p* < 0.05), and IL-1β (10-fold, *p* < 0.01) on day 21. At the same time point, anti-inflammatory cytokines, such as IL-10 (40-fold, *p* < 0.005) and TGF-β1 (12-fold, *p* < 0.005), were also significantly overexpressed. Conversely, in the pathological cells, among the investigated pro-inflammatory cytokines, only IL-6 (2-fold, *p* < 0.05) and IL-1β (30-fold, *p* < 0.05) were the most expressed on day 21, as well as the anti-inflammatory cytokine IL-10 (30-fold, *p* < 0.05) (Figure 7).

### 2.8. Healthy and Pathological hTSPCs Secrete Inflammatory Cytokines Differently

**Figure 8:**
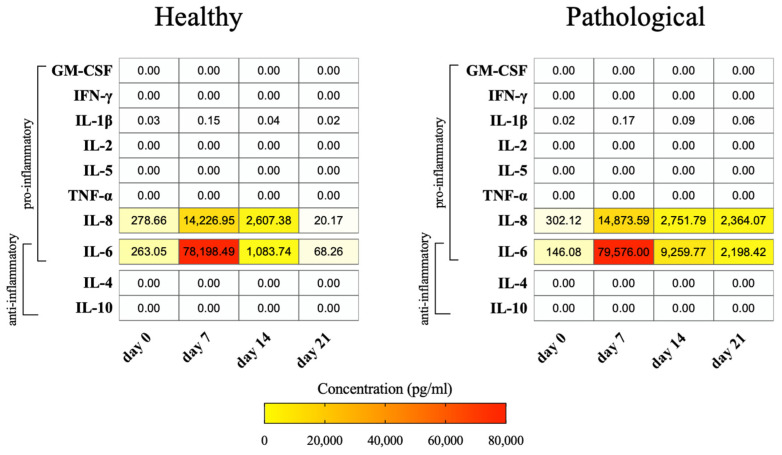
Pro-inflammatory and anti-inflammatory cytokine measurement in the culture medium of healthy and pathological *h*TSPCs. Cytokine levels were detected in cellular supernatants throughout the 3D culture under perfusion on days 0, 7, 14, and 21 using an immunobead-based multiplex assay. Data are reported as a heatmap from white (lowest value, 0 pg/mL of concentration) to red (highest value). The heatmap was made using Prism software (v. 9.0, GraphPad Software, LLC, San Diego, CA, USA).

Cytokine secretion was also investigated by analyzing the culture medium supernatants of healthy and pathological *h*TSPCs on days 0, 7, 14, and 21 using an immunobead-based multiplex assay (Figure 8). Among the 10 cytokines studied, only IL-8 and IL-6 were significantly high in the culture medium of both cell types starting from day 0, reaching a peak on day 7 and decreasing on day 14. Interestingly, we observed that although healthy cells reduced the secretion of inflammatory cytokines below the basal level (day 0) by day 21, these cytokines were still detectable at significant levels in the culture medium of pathological cells. All the other investigated cytokines were not present in the culture medium supernatants.

## 3. Discussion

Tendons connect muscles to bones and enable movement or joint stabilization. Lesions and inflammation can occur in tendons because of mechanical stress, ageing, or a genetic predisposition [46]. The inability of the tendon to self-repair and the inefficiency of current treatment regimens have sparked the exploration of alternative treatment strategies [47]. Tissue engineering and regenerative medicine approaches are promising avenues for nonsurgical treatment presently being explored [48]; they mainly consist of applying growth factors, singly or in combination, with stem cells in native or genetically modified form at the site of tendon damage [48], to try to regain the original tissue or organ structure and function. On the other hand, the principles applied to the development of tissue-engineered constructs can be used as the foundational concepts to engineer relevant in vitro models, to unravel fundamental research questions about physiological or pathological phenomena, keeping in mind their intrinsic limitations [49].

This study aimed to develop an in vitro model to explore tenogenic processes. For this purpose, we utilized a dynamic culture system integrated with a 3D fibrin scaffold, populated with either healthy or diseased *h*TSPCs. The methods used for isolating both healthy and pathological *h*TSPCs, as well as their characterization, covering aspects such as the immunophenotype, multipotency potential, proliferation rate, morphology, and gene expression, have been thoroughly detailed in prior research [40]. Building upon our previous findings and in line with other studies on human tendon tissues [10,11], we verified the stem cell properties of both healthy and diseased hTSPCs by examining their immunophenotype. This analysis confirmed positivity for the mesenchymal stem cell markers (CD73, CD90, and CD105) and negativity for the hematopoietic markers (HLA-DR, CD34, CD14), as per the ISCT guidelines [50] (Figure 1b).

Then, we created a 3D fibrin culture bioengineered with either healthy or pathological *h*TSPCs (Figure 1c). Even if the tendon ECM is mainly composed of hierarchically organized fibrous collagen units, fibrin gels have been demonstrated to exhibit improved structural, biological, and mechanical properties, compared to collagen gels, in cell-based tendon tissue engineering constructs [27]. Based on our previous investigation [41], a fibrinogen concentration of 50 mg/mL was chosen to build the scaffold, because it showed the ideal porosity and fiber interconnectivity to allow good nutrient exchange (Figure 1a). This was confirmed by excellent cell viability up to 21 days, for both cell types (Figure 2). To ensure proper oxygen and metabolite mass transfer into the 3D culture, scaffolds were placed within the culture chambers of a perfusion bioreactor, in which the medium was re-circulated at a constant flow rate of 1 mL/min (Figure 1c). The velocity of the medium can induce shear stress, potentially influencing cell behavior by acting as a mechanical cue. To eliminate any impact of shear stress on cell differentiation and development, the study deliberately selected an extremely low flow rate (1 mL/min). Under these conditions, the FEM analyses confirmed a laminar flow and a uniform velocity distribution within the culture wells (2.95 × 10^−4^ m/s), with a similar order of magnitude to the mean value estimated through the 3D culture system (3.42 × 10^−4^ m/s) (Figure 1d). On the other hand, the relatively low flow rate adopted was sufficient to facilitate active oxygen and mass transfer within the 3D culture, as previously described [44]. Indeed, it is to be considered that, despite its relatively avascular appearance, the tendon is more oxygen-dependent than other joint tissues, such as cartilage, and requires enhanced perfusion during repair [51]. Furthermore, to add a higher level of complexity to the in vitro model, the perfused medium was supplemented with human TGF-β1 at 20 ng/mL, following the data in the literature [52]. Indeed, even if it has been described as one of the most potent profibrogenic factors during the tendon healing process, it has been demonstrated that TGF-β1 blockage fails to effectively enhance tendon healing, because of its key role in promoting tendon cell migration, proliferation, and differentiation [53].

Healthy and pathological *h*TSPCs, cultured in the 3D fibrin scaffold, displayed upregulation of tendon-related markers, such as Tenascin-C (TNC), Scleraxis-A (SCX-A), and Tenomodulin (TNMD) (Figure 3). TNC belongs to a highly conserved family of oligomeric glycoproteins organized in the ECM of vertebrate organisms and its expression suggests tenogenesis in vitro [54]. We found a sustained and significant increase in TNC gene expression over the culture time for both healthy and pathological cells, suggesting the activation of tenogenic events in our culture conditions (Figure 3).

The transcription factor SCX-A serves as a specific marker for both tendon/ligament progenitor cells and differentiated cells during the initial stages of tendon/ligament lineage specification. It is highly expressed throughout tenogenesis, influencing both cell differentiation and extracellular matrix (ECM) organization [55]. Additionally, SCX-A regulates the expression of TNMD, a type II transmembrane protein specifically present in hypovascular connective tissues like tendons, which acts as a phenotypic marker for tendon development [56]. The upregulated gene expressions of SCX-A and TNMD confirmed that our culture system supported the tenogenic events of both healthy and diseased *h*TSPCs (Figure 3). However, we noticed a lower upregulation of the TNMD gene in pathological cells (25-fold), compared to healthy ones (40-fold) (Figure 3), and a lower level of Scleraxis and Tenomodulin protein expression in diseased *h*TSPCs, in contrast with healthy *h*TSPCs (Figure 4 and Figure 5). These findings are corroborated by in vivo studies reporting that Scx-/Tnmd-knockout mice exhibit impaired healing [57,58]. To the best of our knowledge, the difference in Scleraxis and Tenomodulin expression by TSPCs derived from healthy or injured tissues has never been investigated, but it is plausible to hypothesize that pathological TSPCs may exhibit defective expression of these proteins.

Tenomodulin is also involved in collagen fibril maturation [59]. The most abundant form of collagen in the ECM of tendons is type I. Type I collagen fibrils are stiff structures that provide the tendon with mechanical durability and strength. Type I collagen is always associated with another fibrillar collagen, type III collagen, whose fibrils are thinner than type I fibrils. During the healing process in the tendon, a randomly oriented initial network of mostly type III collagen is formed at the wound site. Over time, this granulation tissue is replaced by a stronger, better-aligned network of type I collagen. Thus, type III collagen is generally associated with scar tissue and injury [60]. Indeed, a higher amount of type III collagen deposition in the ECM of pathological *h*TSPCs was found, compared to healthy cells (Figure 5). Moreover, we observed a decrease in type I collagen expression after 14 days, but it was characteristic only of pathological cells under our culture conditions. Treatment with TGF-β1 stimulated the production and accumulation of type I collagen fibers, but, compared to healthy cells, pathological cells appeared to have impaired type I collagen turnover, leading to significant ECM disorganization (as seen in Figure 4).

Furthermore, we analyzed the ratio of type I/type III collagen, which in tendons has been shown to decrease with increased pathology [60]. These data are consistent with our findings, showing a higher type I/type III collagen ratio in healthy *h*TSPCs (>1), compared to pathological *h*TSPCs (<1), throughout the 3D culture, except for day 14 (Figure 6a). At this time point, the pathological cells exhibited a significant increase in type I collagen production (Figure 4), with a type I/type III collagen ratio > 1.5, reaching the same value measured for healthy cells (Figure 6a). In this sense, our 3D dynamic culture conditions plus the growth factor-supplemented medium seemed to positively influence the differential expression of collagen subtypes by healthy and pathological *h*TSPCs.

Inflammation is a pivotal process both in normal and pathologic tendon healing. However, excessive or disrupted inflammatory responses lead to scar-like tendon healing or pathology [61,62]. Animal studies showed that TSPCs play an important role in regulating inflammation during the healing of acute tendon injuries [63]. Unfortunately, to date, the understanding of the inflammation responses and related regulatory roles of *h*TSPCs, obtained from both healthy and pathological tissues, is limited. In this study, we selected several pro-inflammatory and anti-inflammatory cytokines to be monitored at gene and protein levels. According to our findings, *h*TSPCs demonstrated active involvement in regulating the expression of cytokines, but their behavior varied depending on the healthy or pathological status of the tissue source. Pathological *h*TSPCs demonstrated a decreased overall cytokine expression when compared to healthy *h*TSPCs **(**Figure 7), but the culture medium of both cell types showed significantly elevated levels of secreted IL-8 and IL-6 (Figure 8). Indeed, IL-6 is a key pro/anti-inflammatory cytokine that exerts a pleiotropic role in innate and adaptive immunity, as in tendon healing and pathology [63], contributing to the inflammatory cascade, along with IL-8, through a defined IL-6/IL-8 ratio [64]. Variations were noted in the secretion of IL-8 and IL-6 by pathological cells, with sustained high levels of both cytokines on day 21, a phenomenon not observed in healthy cells (Figure 8), suggesting a different secretory profile for *h*TSPCs under pathological conditions. This finding confirmed our previous data reporting a characteristic overexpression of pro-inflammatory cytokines in pathological cells, compared to healthy ones [39]. This is consistent with the fact that the tendon stem cell population is actively involved in inflammation and tendon healing after injury and may play a crucial role in resolving pathological conditions [65]. Based on our results, we hypothesize that pathological cells exhibit an aberrant inflammatory response compared to healthy cells, with potentially delayed resolution times. However, it would be valuable to conduct an in-depth investigation of the entire gene expression profile of both healthy and pathological stem cells in this context.

Unexpectedly, we also observed a complete absence of the anti-inflammatory cytokines IL-4 and IL-10, even after 21 days of culture (Figure 8). This could be attributed to the complex sequence of events that characterize tendon healing, combined with the limitations of our culture conditions. The tendon biopsies from which we harvested *h*TSPCs were most likely in the acute inflammatory phase following injury. After tendon damage, a specific sequence of events unfolds to promote healing and repair. These events occur quickly after tendon failure, creating a pro-inflammatory environment within 2–3 days post-injury. IL-6 is the primary pro-inflammatory cytokine present during this phase [66], as confirmed by our results. However, IL-6 also functions as an anti-inflammatory regulator, especially during the subsequent proliferative phase (2–6 weeks post-injury), which is marked by the resolution of inflammation and the onset of healing [66]. Indeed, we observed higher levels of IL-6 in the supernatant of pathological cells compared to healthy cells after 21 days. It is also worth noting that although TGF-β1 is well-known for its anti-inflammatory properties [32], the treatment with TGF-β1 might have had a slight impact on the secretion of other anti-inflammatory cytokines under the conditions being investigated.

In conclusion, to our knowledge, this is the first study using a 3D culture system incorporating both healthy and pathological *h*TSPCs, representing a novel advancement in the tissue engineering field. In this in vitro model, pathological cells exhibited distinct behavior compared to healthy cells, particularly in terms of tenogenic and inflammatory responses. Indeed, despite numerous in vivo and in vitro studies suggesting the active role of TSPCs in tendon injuries and the related inflammation process, the biological mechanisms governing stem cell differentiation and tendon regeneration remain inadequately understood. In this regard, our in vitro biomimetic 3D model shows significant potential to improve the understanding of *h*TSPCs’ involvement in the tenogenic process under both healthy and pathological conditions and offers promising avenues for further investigation into their behavior in tendon regeneration processes.

## 4. Materials and Methods

### 4.1. Collection of Human Biopsies

A total of four healthy semitendinosus (males; 25, 28, 51, and 73 years old) and four tendinopathic tendons (males; 28, 43, 51, and 63 years old) were obtained, after informed consent was given, according to protocols approved by the Institutional Review Board of “San Giovanni di Dio e Ruggi D’Aragona Hospital” (Salerno, Italy) (Review Board prot./SCCE n. 151 achieved on 29 October 2020). Healthy tendon samples were collected from non-suitable tissue parts of semitendinosus autologous transplants after reconstruction of the anterior cruciate ligament, whereas tendinopathic tendon samples were harvested from patients undergoing surgery following Achilles tendon trauma. The tendon composition should not greatly differ based on the anatomical site, as long as the tendons and ligaments are not mixed [67]. The presence of comorbidities and any previous or concurrent anterior cruciate ligament/Achilles tendon disease were considered as exclusion criteria.

### 4.2. The hTSPC Harvesting and Characterization

The *h*TSPCs were harvested from both healthy and tendinopathic tissue samples, using a method described elsewhere [39]. The cells were cultured in alpha-minimum essential medium (α-MEM, Corning Cellgro, Manassas, VA, USA), supplemented with 1% Glutagro™, 1% penicillin/streptomycin (Pen/Strep), and 10% Fetal Bovine Serum (FBS), and incubated at 37 °C in an atmosphere of 5% CO_2_ and 95% relative humidity. After the extraction, the *h*TSPCs were used for immunophenotype analysis and for 3D culture preparation at passage 2 to avoid phenotype drift.

### 4.3. Flow Cytometry and Gating Strategy of hTSPCs

Healthy and pathological *h*TSPCs were harvested and counted, and 1 × 10^5^ cells were incubated at room temperature for 20 min with the following directly conjugated mouse anti-human antibodies: CD34-PE, CD90-FITC, CD105-PE, HLA class II-FITC, CD14-PC7 (all from Beckman Coulter, Fullerton, CA, USA), and CD73-APC (Miltenyi Biotec, Gladbach, Germany). After incubation, the samples were washed twice with 1× phosphate buffered saline (PBS; Corning Cellgro) and resuspended in the same buffer for flow cytometry analysis. The samples were then analyzed using a BD FACSVerse flow cytometer (Becton Dickinson, BD, Franklin Lakes, NJ, USA), equipped with two lasers (blue 488 nm and red 628 nm). Compensation settings were established using single-color controls for each fluorochrome, along with an unstained sample as a negative control for setting the photomultiplier tube (PMT) voltages. Consistent PMT voltages were applied across all samples, with a minimum of 30,000 events recorded per sample. The Kaluza software (v.2.1, Beckman Coulter, Brea, CA, USA) was used for the compensation and flow cytometric analysis following acquisition. The *h*TSPCs were initially identified using linear parameters (the forward scatter area [FSC-A] versus the side scatter area [SSC-A]) and doublets were excluded (FSC-A versus FSC-H). The expression levels of each marker on single cells were visualized using histograms, with an unstained sample serving as the negative control.

### 4.4. Fibrin Scaffold Drying, FE-SEM, and Pores Analysis

Cylindrical 3D scaffolds were created by mixing fibrinogen (50 mg/mL, Sigma-Aldrich, St. Louis, MO, USA), α-aprotinin (15,600 U/mL, Sigma-Aldrich), α-MEM (Corning, NY, USA), and thrombin (100 U/mL, Sigma-Aldrich). The mixture was incubated at 37 °C for 30 min to allow the fibrinogen to polymerize. Afterwards, the samples were fixed overnight in 4% paraformaldehyde (PFA) at 4 °C. Following fixation, the samples were dehydrated through a series of ethanol–water solutions, with gradually increasing ethanol concentrations, with each step lasting 10 min. The samples were then dried using dense carbon dioxide at 200 bar and 38 °C for 4 h, following previously established methods [41,42]. Once frozen in liquid nitrogen and fractured with a needle, the samples were mounted on double-sided adhesive carbon tape fixed to an aluminum stub. A 250 Å thick gold layer was applied using a sputter coater (mod.108 A; Agar Scientific, Stansted, UK), to prepare the samples for observation. The scaffold’s internal structure was analyzed using field emission scanning electron microscopy (FE-SEM, mod. LEO 1525; Carl Zeiss, Oberkochen, Germany). Feret’s diameter of the fibrin pores was measured using ImageJ software (rel.1.52p, National Institutes of Health, Bethesda, MD, USA), and the nearest neighbor distance and average wall thickness were determined using an external ImageJ plug-in (NND), according to a standardized protocol [68].

### 4.5. Assembly of 3D Bioengineered Scaffolds and Perfusion Culture System

The cylindrical 3D scaffolds were created by combining fibrinogen (50 mg/mL, Sigma-Aldrich), α-aprotinin (15,600 U/mL, Sigma-Aldrich), and α-MEM (Corning, NY, USA), containing 2 × 10^6^ cells/mL (either healthy or pathological). The mixture was distributed into different wells in a 96-well plate, followed by the addition of thrombin (100 U/mL, Sigma-Aldrich). The samples were then incubated at 37 °C for 30 min to facilitate fibrinogen polymerization. These 3D bioengineered scaffolds were subsequently placed on the culture plate of a perfusion bioreactor (Figure 1c). The bioreactor consisted of two custom-made multi-well plates, machined from poly (methyl methacrylate) (PMMA, Altuglas^®^ CN 100 10000, Altuglas International, La Garenne-Colombes Cedex, France), a biocompatible material used for biomedical applications [69]. Each plate featured two openings for inserting silicon tubes (Tygon^®^, VWR, Milan, Italy) to allow a medium flow at a constant rate of 1.0 mL/min, maintained by peristaltic pumps [45]. This bioreactor system was operated within a standard cell culture incubator, with medium velocity calculated based on previous finite element modelling (FEM) simulation data [44,45].

### 4.6. Live and Dead Assays

Cell viability was detected using fluorescence live and dead assays immediately after preparation (Day 0) and at each time point (days 7, 14, and 21). Calcein AM solution (Cat. No C1359, Sigma-Aldrich) was used to stain live cells, while cell membrane-impermeable ethidium homodimer I solution (Cat. No E1903, Sigma-Aldrich) was used for the nuclei of dead cells. The cells were incubated for 1 h at 37 °C, then washed in PBS 1× and images were captured at 4× magnification using a fluorescence microscope (Eclipse Ti Nikon Corporation, Tokyo, Japan). The signal intensity was quantified using ImageJ software. Original RGB images were converted into an 8-bit (greyscale) format and the tagged area intensities were expressed as the mean value of the pixel intensity within a range from 0 (dark) to 255 (white), as previously reported [70].

### 4.7. RNA Isolation and Gene Expression Profiling

The gene expression analysis of tenogenic markers, including SCX-A, DCN, COL1A1, COL3A1, TNC, and TNMD (Bio-Rad, Foster City, CA, USA), as well as cytokines including as IL-6, TNF, IL-12A, IL-1β, IL-10, and TGF-β1 (sequences detailed in Table 1), was conducted using reverse transcription quantitative polymerase chain reaction (RT-qPCR) tests. The total RNA was extracted from 3D dynamic cultures at each time point, utilizing QIAzol Lysis Reagent (Qiagen, Hilden, Germany), chloroform (Sigma-Aldrich), and the RNeasy Micro Kit (Qiagen). For each sample, 1 μg of RNA was reverse transcribed into cDNA using the iScript™ cDNA synthesis kit (Bio-Rad, Foster City, CA, USA). The relative gene expression was analyzed using a LightCycler^®^ 480 instrument (Roche, Basel, Switzerland) with SsoAdvanced™ Universal SYBR^®^ Green Supermix (Bio-Rad). The specificity of the PCR products was verified through melting curve analysis. The gene expression data were normalized to the reference gene, glyceraldehyde-3-phosphate dehydrogenase (GAPDH), using the geNorm method [71] and the CFX Manager software (Version 3.1, Bio-Rad, Foster City, CA, USA) (M < 0.5). Fold changes in the gene expression were calculated using the 2^−ΔΔCt^ method, presented as relative levels compared to day 0 (*h*TSPCs immediately after incorporation into the fibrin scaffold). All the experiments were conducted in biological quadruplicates (n = 4), with each in technical triplicates.

### 4.8. Immunohistochemical Assay

Fibrin scaffolds were fixed in 4% PFA for 2 h at RT, cryo-protected in 30% sucrose (4 °C, overnight), included in the optimal cutting temperature (OCT) compound, and cut into slices of 10 μm thickness using a cryostat (CM 1950, Leica, Wetzlar, Germany). The slices were permeabilized with 0.1% Triton X-100 for 10 min and blocked with BSA 1% solution for 1 h. The samples were then stained for Tenomodulin (1:100; Abcam, Cambridge, UK), type I collagen (1:100; Sigma-Aldrich), Scleraxis-A (1:100, Abcam), and type III collagen (1:50; Sigma-Aldrich), incubated overnight at 4 °C, and subsequently incubated for 1 h at RT with Alexa Fluor^™^ 488 goat anti-rabbit IgG (1:500; Thermo Fisher Sci., Waltham, MA, USA), and VectaFluor™ anti-mouse IgG DyLight 594^®^ kit (1:500; Vector laboratories, Newark, CA, USA). The cell nuclei were counterstained using DAPI. Separate images were acquired at 20× magnification using identical settings in terms of the light intensity, exposure time, and gain, using a Leica laser scanning confocal microscope (mod. TCS SP5; Leica Microsystems, Wetzlar, Germany).

### 4.9. Hematoxylin and Eosin Staining

Scaffold slices (10 μm thickness) were washed for 5 min in water and incubated with Hematoxylin (1 min) and Eosin (5 min), then dehydrated using an increasing ethanol gradient (75–95–100%) and cleared in xylene for 5 min. Sections were mounted using the Eukitt (Sigma-Aldrich) mounting medium. Images were acquired at 20× magnification using an Olympus microscope BX53, equipped with a ProgRes SpeedXT core five camera.

### 4.10. Sirius Red Staining

The Picro-Sirius Red Staining Kit (Polysciences, Inc., Warrington, PA, USA) was used to perform collagen fiber staining. Sections with a thickness of 10 μm were stained in Hematoxylin for 8 min, washed in water for 2 min, immersed in phosphomolybdic acid for 2 min, washed in water for 2 min, dipped into Picro-Sirius Red F3BA Staining fluid for 60 min, and then into a HCl 0.1 M solution for 2 min. The sections were dehydrated in solutions with an increasing ethanol gradient (70–75–95–100%) and finally immersed in xylene for 5 min. The samples were mounted using the Eukitt medium and dried under a chemical hood for 30 min. The Picro-Sirius Red brightfield and cross-polarized light images were acquired at 20× magnification with a Leica DMD108 polarization microscope.

### 4.11. Detection of Cytokine Release

An evaluation of the levels of 10 cytokines/chemokines/inflammatory molecules [GM-CSF (Granulocyte-Macrophage Colony-Stimulating Factor), interferon (IFN)-γ, IL-1β, IL-2, IL-4, IL-5, IL-6, IL-8, IL-10, and Tumor Necrosis Factor (TNF)-α] in the 3D culture supernatants was carried out using the Human Cytokine Magnetic 10-Plex Panel (Invitrogen, Camarillo, CA, USA), according to the manufacturer’s protocol. The plate was read using the Luminex MAGPIX^®^ platform. The xPONENT^TM^ software (v. 4.2, Luminex Corporation, Austin, TX, USA) was employed for data analysis.

### 4.12. Statistical Analysis

Statistical analysis of the obtained data was performed using Prism software (v. 9.0, GraphPad Software, LLC, San Diego, CA, USA). The results, collected from multiple experiments (n = 4), are presented as the mean ± standard deviation (SD). To assess the statistical significance among the independent groups, Student’s *t*-test and the Mann–Whitney test were used [72,73]. A *p*-value < 0.05 was considered statistically significant.

## Figures and Tables

**Table 1:** Primer sequences for cytokine gene expression analysis.

Target	Gene Bank Accession Number	Sequences	Product Size	Primer Efficiency
IL-6	NM-000600.5	Fwd: ACTTGCCTGGTGAAAATCAT Rev: CAGGAACTGGATCAGGACTT	135	106
TNF	NM-000594.4	Fwd: GCCCATGTTGTAGCAAACCC Rev: TATCTCTCAGCTCCACGCCA	97	105
IL-12A	NM-000882.4	Fwd: TCAGAATTCGGGCAGTGACT Rev: AGTCCCAQTCCTTCTTTCCCC	163	110
IL-1β	NM-000576.3	Fwd: GGAGAATGACCTGAGCACCT Rev: GGAGGTGGAGAGCTTTCAGT	185	110
IL-10	NM-000572.3	Fwd: AAGACCCAGACATCAAGGCG Rev: AATCGATGACAGCGCCGTAG	85	110
TGF-β1	NM-000660.7	Fwd: GCACTCGCCAGAGTGGTTAT Rev: AAGCCCTCAATTTCCCCTCC	81	95

## Data Availability

All data generated or analyzed during this study are included in this manuscript.
